# Screening of genes interacting with high myopia and neuropsychiatric disorders

**DOI:** 10.1038/s41598-023-45463-y

**Published:** 2023-10-26

**Authors:** Yang Liu, Yang Liu, Wen Zhang, Zhong-Qi Xue, Fang-Xia Zhang, Wei-Gang Xu, Wen-Juan Zhuang

**Affiliations:** 1 People’s Hospital of Ningxia Hui Autonomous Region (Ningxia Eye Hospital), Yinchuan, China; 2 Department of Ophthalmology, Affiliated Hospital of Qingdao Binhai University, Qingdao, China

**Keywords:** Biological techniques, Computational biology and bioinformatics, Genetics

## Abstract

Clinical studies have demonstrated an association between high myopia (HM) and neuropsychiatric disorders; however, the underlying mechanism of the association is not clear. We used whole exome sequencing (WES) in combination with the Genetic Variants Classification Criteria and Guidelines published by the American College of Medical Genetics (ACMG) and bioinformatics analysis to clarify the interrelationship between candidate genes. Causative genes for ocular diseases (45.38%) followed by neuropsychiatric disorders (22.69%) accounted for the highest proportion of genes that exhibited high pathogenicity in HM patients were found. Four pathogenic gene mutations were identified according to ACMG guidelines: c.164_165insACAGCA and c.C1760T in *POLG*, c.G1291A in *COL5A1*, and c.G10242T in *ZNF469*. Three causative genes for neuropsychiatric diseases, *PTPRN2, PCDH15* and *CDH23*, were found to fall at the HM locus. The above results suggest that these genes may interact in high myopia and neuropsychiatric diseases.

## Introduction

High myopia (HM), a common ophthalmic disease, is an important global public health problem, especially in Southeast Asia^1,2^. HM often leads to a series of complications (such as retinopathy and glaucoma) that can cause blindness. Therefore, development of ocular complications is the key concern during clinical treatment of HM, while the neuropsychiatric disorders that accompany HM tend to be overlooked^3^.

HM has been shown to be associated with a variety of neuropsychiatric disorders such as anxiety, depression, and cognitive dysfunction^4^. An estimated one-third of the European population with visual impairment is affected by anxiety or depressive symptoms^5^. The estimated prevalence of anxiety or depression in patients with HM in East Asia ranges from 22.0% to 25.9%^6^. Among elderly individuals, the prevalence of cognitive dysfunction in myopic patients was found to be twice as high as that in the individuals with normal vision^7^.

The development of HM is strongly associated with genetic factors as shown by twin and familial aggregation studies^8^. As of today, 25 loci are associated with HM (MYP1-MYP3, MYP5-MYP26), based on whole-exome sequencing (WES) and other tests^9^. Meanwhile, There is a substantial genetic component to most neuropsychiatric disorders, with heritability ranging from 75 to 80%^10^. In conjunction with clinical manifestations associated with high myopia and neuropsychiatric disorders identification of genes that show interaction with both HM and neuropsychiatric disorders is a potential way to explore the genetic mechanism of causation of neuropsychiatric diseases in HM patients^11–13^. In this study, we explored the interaction between HM and neuropsychiatric genes and identified interacting genes in 83 non-syndromic patients with HM from Northwest China.

## Materials and methods

### Study population

Eighty-three sporadic patients with HM were recruited in this study. The study was performed in accordance with the tenets of the Declaration of Helsinki. Written informed consent was obtained from the participants or their statutory guardians, with approval from the institutional review board of the People’s Hospital of Ningxia Hui Autonomous Region, Third Clinical Medical College of Ningxia Medical University. Genomic DNA was extracted from peripheral blood samples. All 83 patients included in this study belonged to the Ningxia Hui Autonomous Region and the clinic information of the patients have been show in Supplementary Table 1. Patients were recruited according to the following inclusion criteria: (1) refractive error worse than or equal to − 6 D and/or axial length (AL) > 26.00 mm; and (2) absence of any other known ocular or systemic disorder.

### Whole exome sequencing and variant filtering

Exome Enrichment V5 Kit (Agilent Technologies, United States) was used for whole exome sequencing as previously described^14^. Sequencing of DNA fragments was conducted on Illumina HiSeq 2000, Analyzers (90 cycles per read). In accordance with the manufacturer's instructions, Illumina libraries were prepared and generated using the Hiseq2000 platform from Illumina, Inc. A set of local realignments, quality control text, and variants were called using the Genome Analysis Toolkit (GATK). As described previously, Sequencing Reads were aligned to the human reference sequence (hg19/GRCH37) using Burrows Wheeler Aligner-Maximum Exact Match (BWA-MEM)^15^. The WES resulted in a mean read depth of 30x. Median coverage of the targeted regions exceeded 95%.

**Figure 1:**
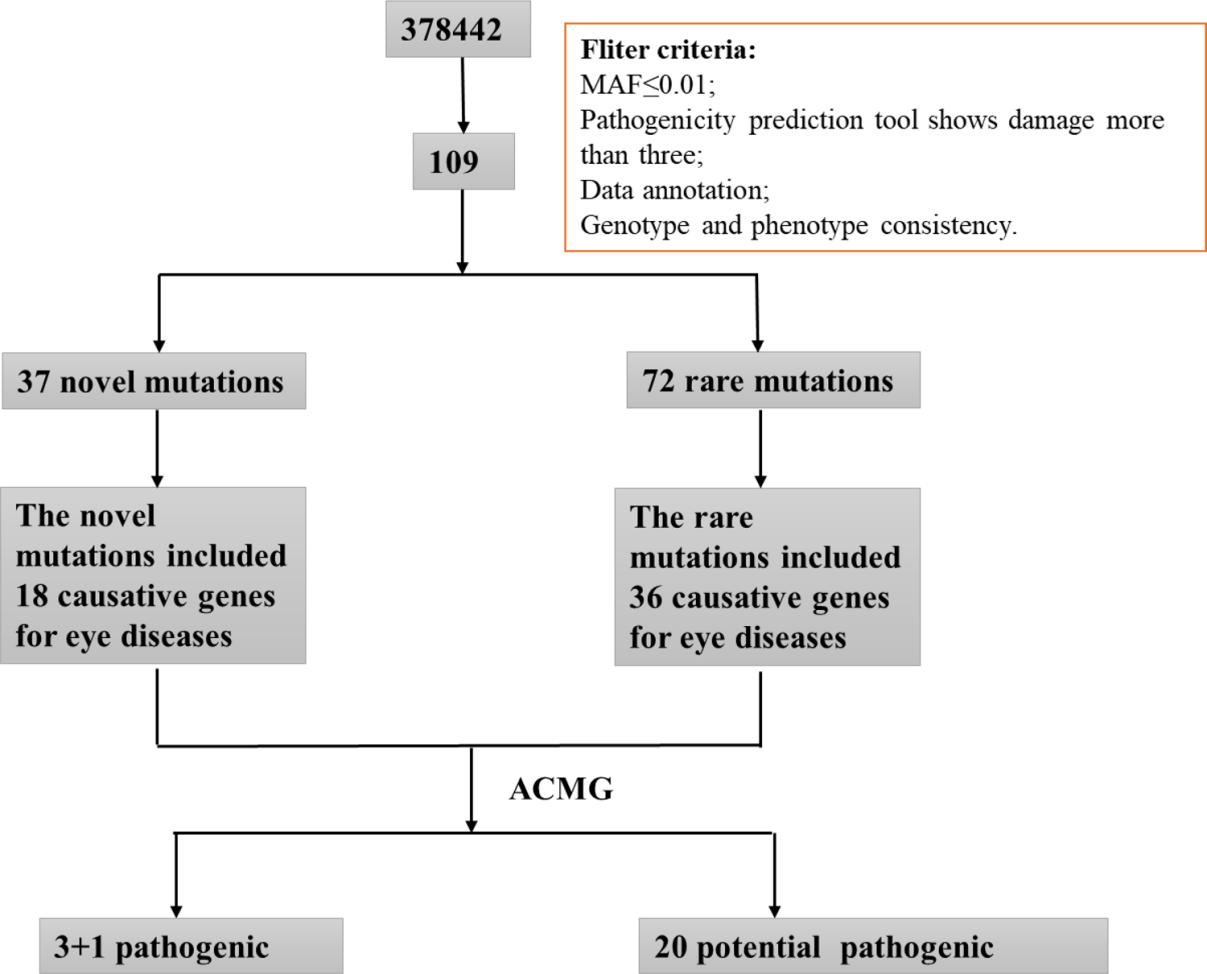
Overview of the screening process. A range of strategies were applied to filter the causative genes for high myopia and neuropsychiatric disorders in 83 sporadic patients. Mutation frequency in populations, Mutation pathogenicity prediction, and ACMG are combined to narrow down the candidate genes and mutations.

The WES results from patients with HM were filtered as follows: (1) Screening of mutations with minor allele frequency (MAF) below 0.01 in the four major human gene frequency databases (1000G, ExAC, ESP6500, and gnomAD); (2) Retention of mutations shown to be Probably Damaged or Damaged by three or more pathogenic prediction software; (3) Comprehensive analysis of strongly pathogenic mutations in patients based on the American College of Medical Genetics (ACMG) guidelines combined with their clinical phenotypes. The screening process is shown in Fig. 1.

All the identified variants were assessed using the following tools. The pathogenicity of gene mutations was predicted using 8 bioinformatics tools: SIFT(http://sift.jcvi.Org), Polyphen2(http://genetics.bwh.harvard.edu/pph2/index.Shtml), LRT(genetics.wustl.edu/jfla), Mutation Taster(https://www.mutationtaster.org/), Mutation Assessor(mutationassessor.org/r3), FATHMM(fathmm.biocompute.org.uk), LR(https://doi.org/10.1093/hmg/ddu733), and Radial SVM(https://doi.org/10.1093/hmg/ddu733). Protein interactions were assessed using String (STRING: functional protein association networks (https://string-db.org/)).

## Results

### Comprehensive analysis of WES results

**Figure 2:**
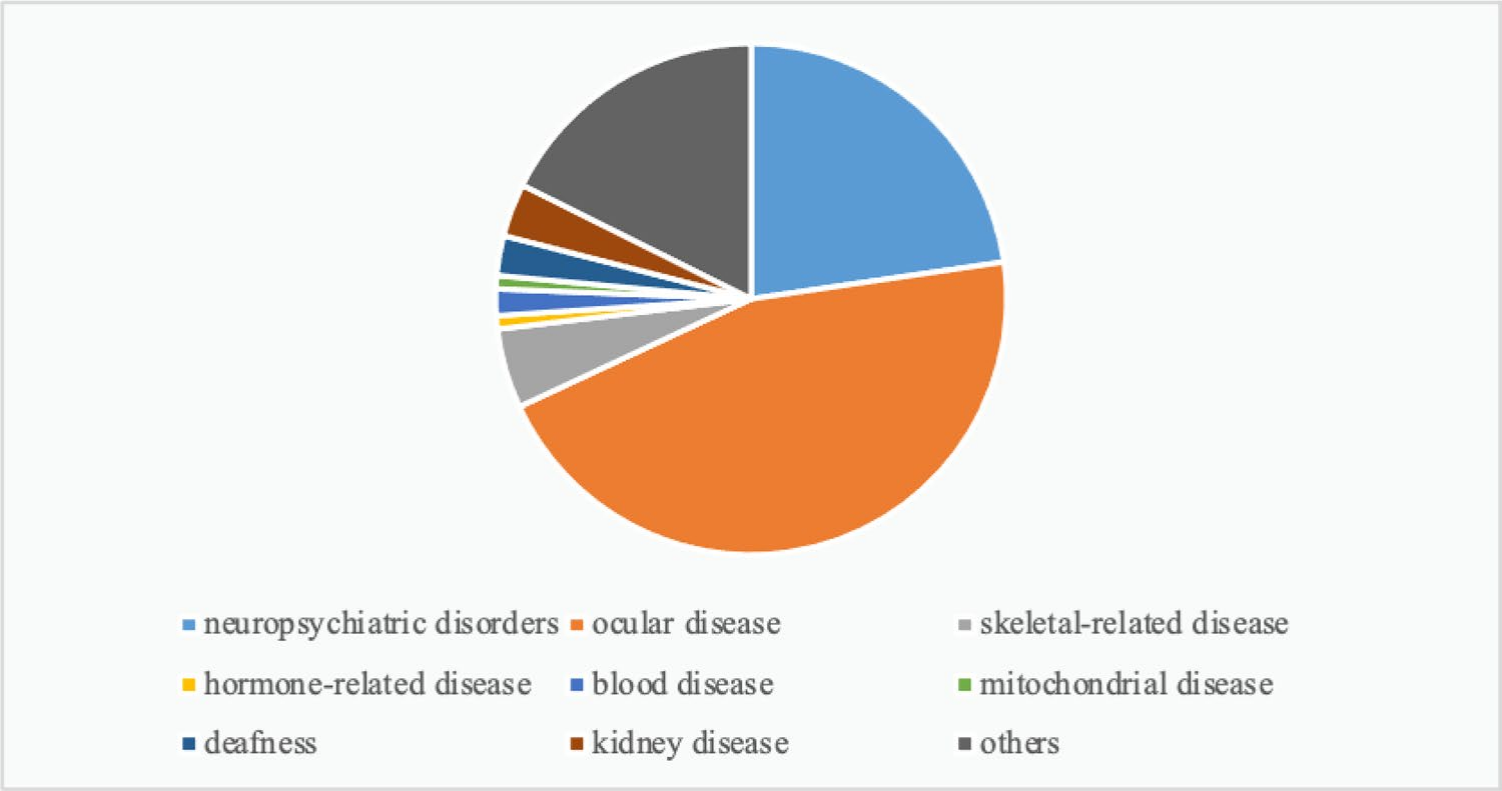
Distribution of strongly pathogenic genes corresponding to various diseases in patients with high myopia. Genes of neuropsychiatric disorders for 22.69%; genes of ocular disease for 45.38%; genes of skeletal-related disease for 5.04%; genes of hormone-related disease for 0.84%; genes of blood disease for 1.68%; genes of mitochondrial disease for 0.84%; genes of deafness for 2.52%; genes of kidney disease for3.36%; genes of others for17.65%.

**Figure 3:**
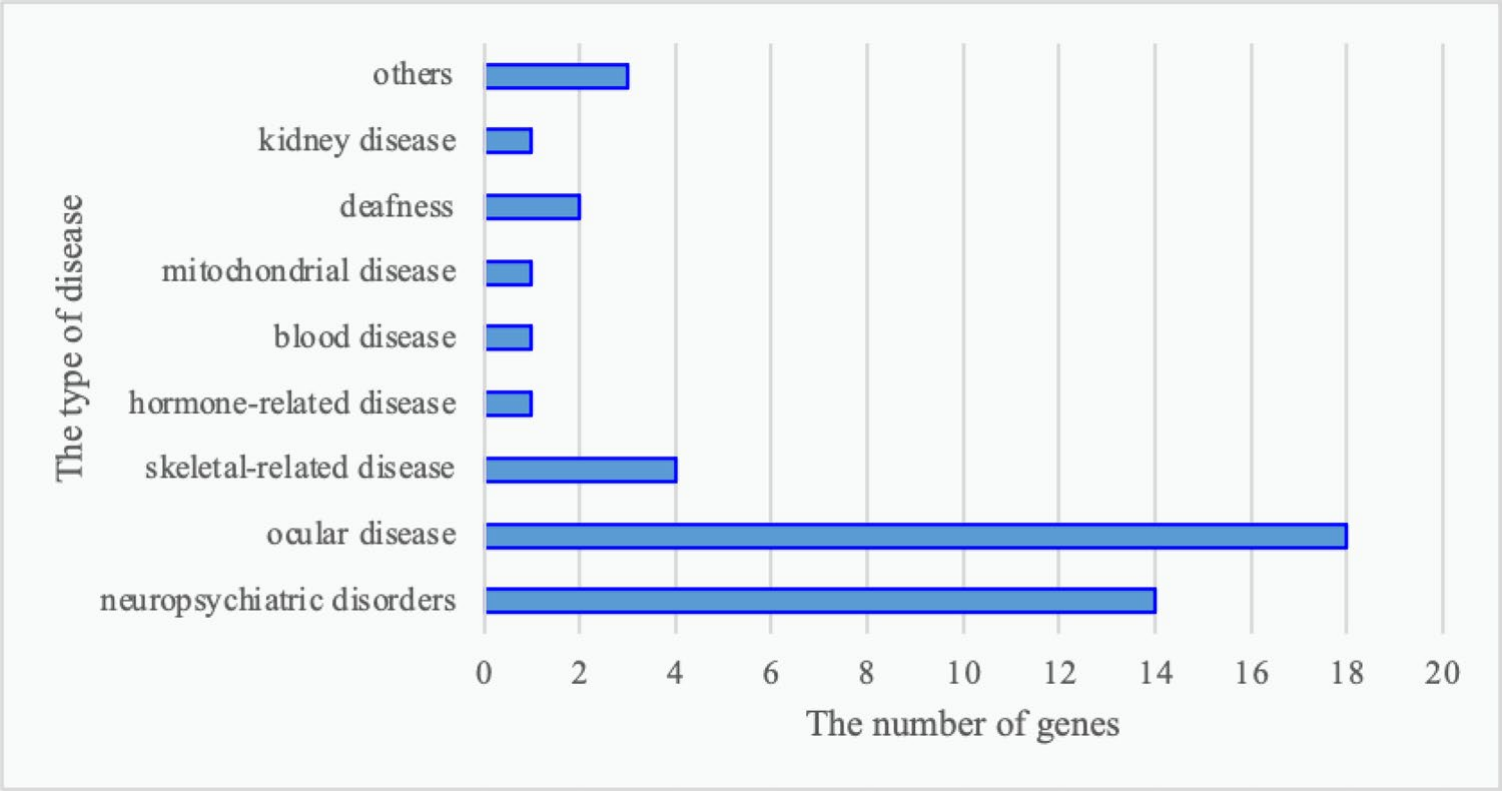
Distribution of disease-related genes among novel mutations. 18 causative genes were included in novel mutations for eye diseases (40%), 14 causative genes for brain diseases (31%), 4 causative genes for skeletal-related disorders (9%), 2 causative genes for deafness (4%), and only 1 causative gene for both hormone-related genes and blood disease (2%).

**Figure 4:**
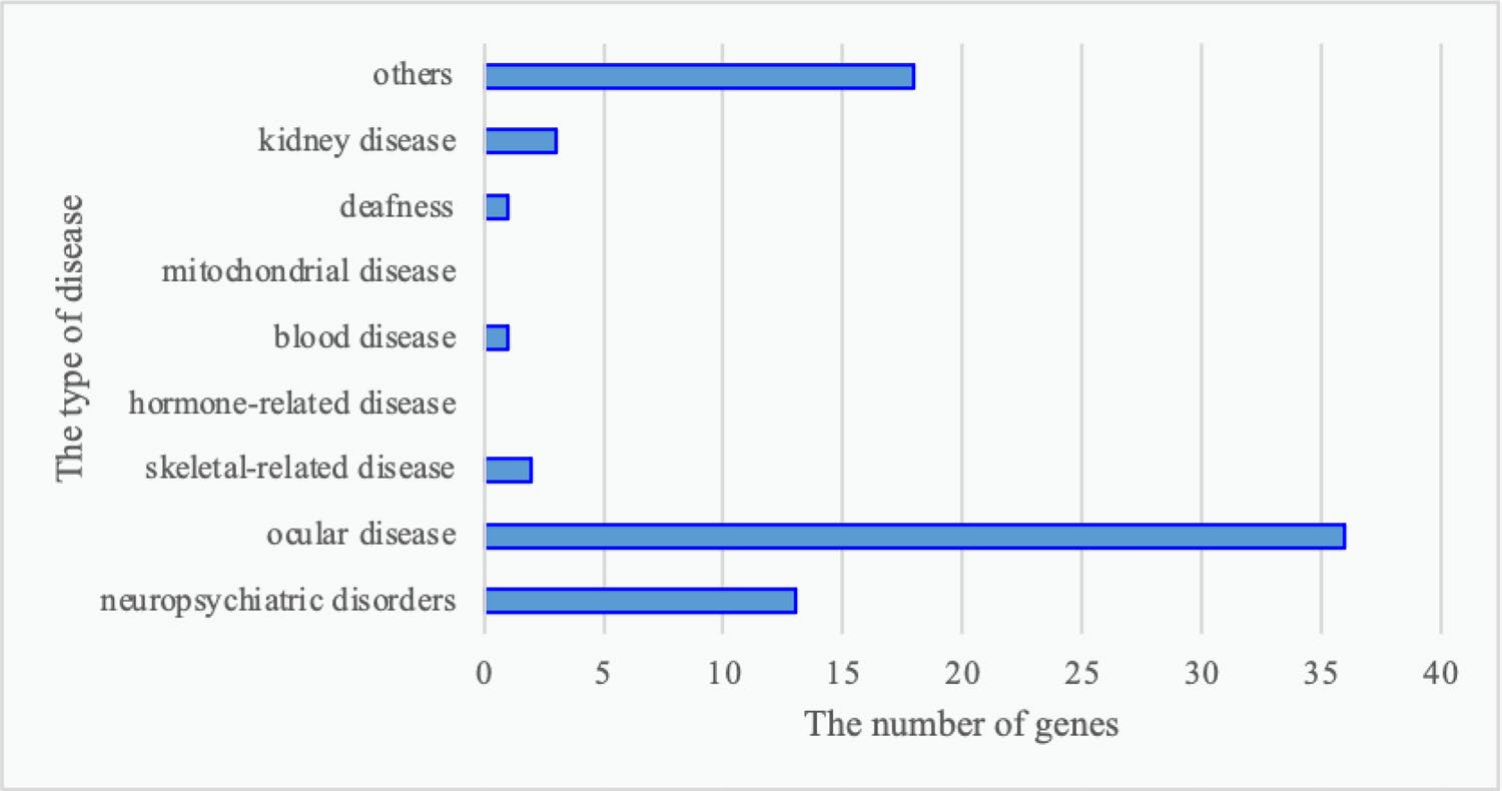
Distribution of disease-related genes among rare mutations. The proportion of rare mutations in various disease-related genes was 36 (49%) for eye disease genes, 13 (18%) for brain disease genes, 3 (4%) for kidney disease genes, 2 (3%) for bone-related genes, and 1 (1%) for both deafness and blood-related genes.

A total of 109 mutations were predicted to be pathogenic by three or more bioinformatics tools; these included 37 novel mutations that have not been reported and 72 rare mutations (Supplementary Tables 2 and 3) (The datasets presented in this study can be found in online repositories. The names of the repository/repositories and accession number(s) can be found below: https://doi.org/10.6084/m9.figshare.19550926.v1). Among these, the strongly pathogenic genes in HM patients were mainly distributed across nine different disease types, with the highest proportion of genes accounting for ocular diseases (45.38%) followed by neuropsychiatric diseases (22.69%) (Fig. 2). After obtaining the proportion of all genes in each disease-related gene, the proportions of various disease-causing genes among the 37 novel mutations and 72 rare mutations were counted. The novel mutations included 18 causative genes for eye diseases (40%), similarly, the proportion of rare mutations in various disease-related genes was 36 (49%) for eye disease genes (Figs. 3, 4). The combined overall data and both partial data indicated that the highest detection rates of mutations in causative genes for ocular and neuropsychiatric disorders were found in patients with HM who had strongly pathogenic genes.

### Analysis of association between causative genes for high myopia and neuropsychiatric disorders

**Figure 5:**
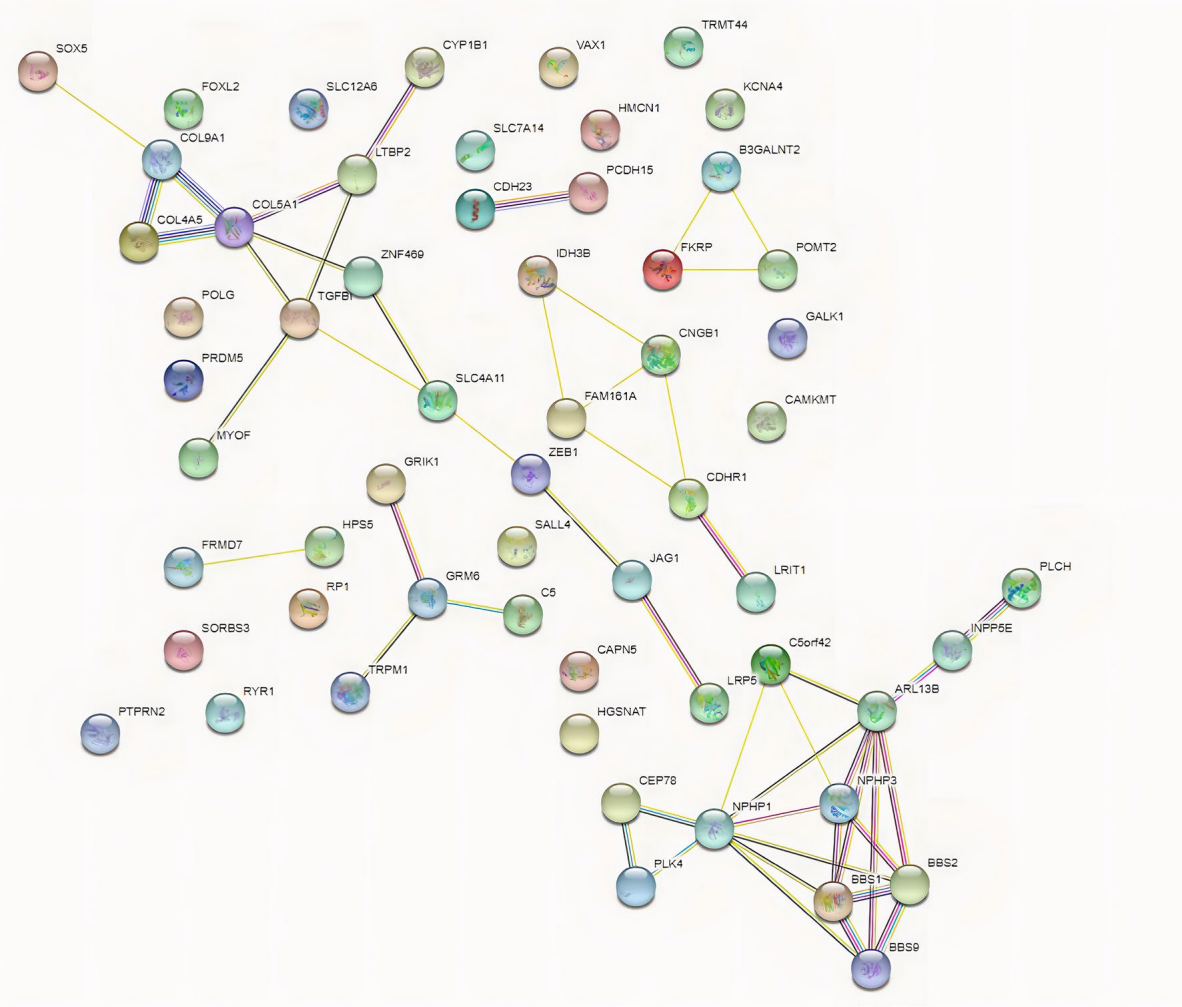
Protein interactions of neuropsychiatric and ocular diseases. One of the gene clusters was *SOX5, COL9A1, COL4A5, COL5A1, LTBP2, CYP1B1, TGFBI, ZNF469, MYOF,* and *SLC4A11*, with direct interactions between *COL9A1, COL4A5*, and *COL5A1*. The other gene cluster was *PLCH, INPP5E, ARL13B, NPHP3, BBS2, BBS1, BBS9, NPHP1, CEP78, PLK4,* and *C5orf42*, with the strongest interactions between *ARL13B, NPHP3, BBS2, BBS1,* and* BBS9.*

The genes related to neuropsychiatric disorders and high myopia were among the 37 novel mutations and 72 rare mutations with strong pathogenicity. The results are summarized in Supplementary Tables 4 and 5. Protein interaction analysis of causative genes for high myopia and neuropsychiatric disorders showed that the genes with strong interactions included two gene clusters One of the gene clusters was *SOX5, COL9A1, COL4A5, COL5A1, LTBP2, CYP1B1, TGFBI, ZNF469, MYOF,* and *SLC4A11*, with direct interactions between *COL9A1, COL4A5*, and *COL5A1*. The other gene cluster was *PLCH, INPP5E, ARL13B, NPHP3, BBS2, BBS1, BBS9, NPHP1, CEP78, PLK4,* and *C5orf42*, with the strongest interactions between *ARL13B, NPHP3, BBS2, BBS1,* and *BBS9* (Fig. 5). It indicates that these genes do not act alone in high myopia or neuropsychiatric disorders, but show multiple interactions with each other.

**Figure 6:**
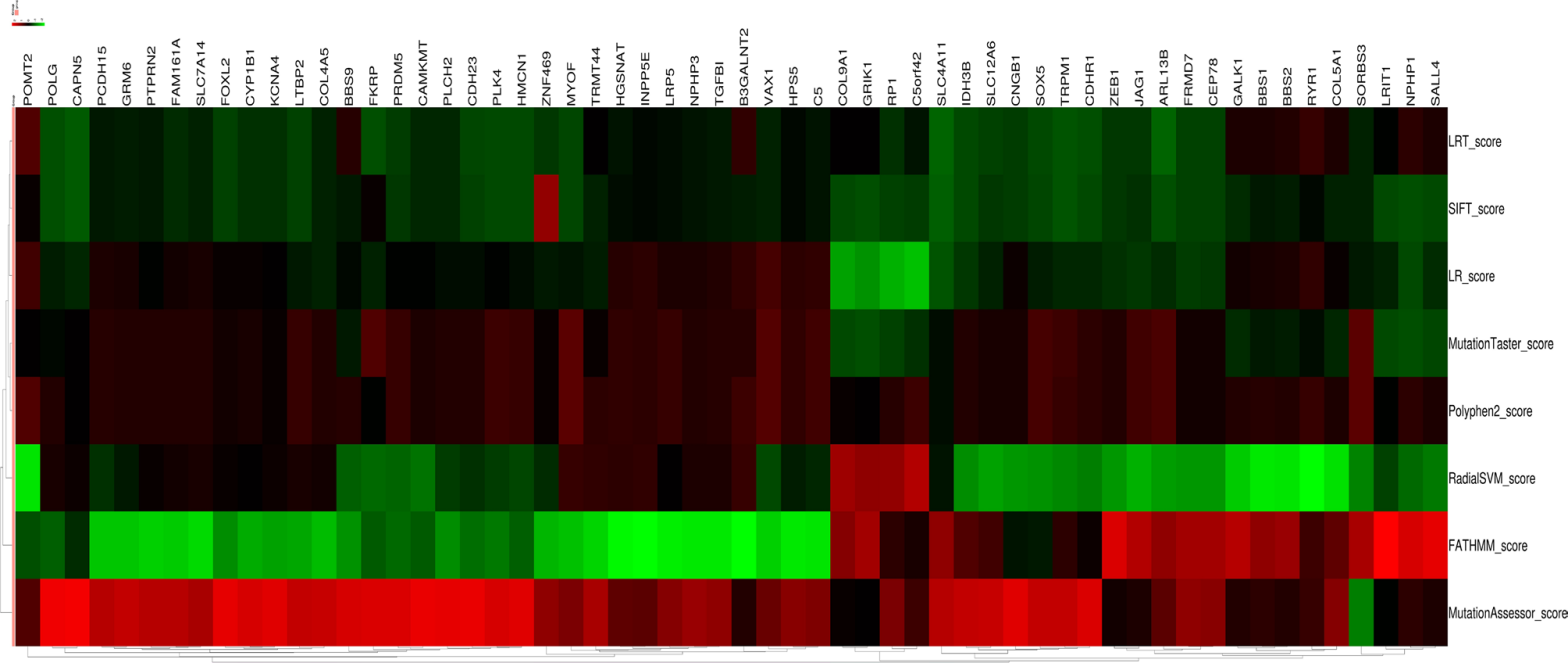
The pathogenicity heatmap of mutations. Different colors represent different degrees of pathogenicity; the darker the color, the greater is the pathogenicity.

We performed pooled analysis of the pathogenicity of all neuropsychiatric disorders-related genes and high myopia-related genes using eight pathogenic prediction software. Two software programs that focus on evolutionary conservation, Mutation Assessor and SIFT, yielded consistent scores for all genes, indicating that these genes have remained extremely conserved at the species level over thousands of years of human genetic progression and that, in turn, the mutations in these genes have a significant impact on the structure and function of human proteins. The consistency of scoring the same group of genes across different software programs collectively improves the accuracy of predicting the pathogenicity of these genes (Fig. 6). In addition, the protein interactions of *ARL13B, NPHP3, BBS2, BBS1,* and *BBS9* gene clusters were all predicted to exhibit high pathogenicity.

The mutations were classified according to the ACMG guidelines. The results showed that c.8815G > A in *HMCN1* and c.541G > A in *SALL4* have both been reported to be benign, combined with the evidence that the allele frequency of the c.8815G > A in *HMCN1* is below the gene frequency threshold in normal populations; c.8815G > A in *HMCN1* and c.541G > A in *SALL4* were determined to be Likely Benign. Another 27 genes were found to be potentially pathogenic, among which four mutations c.164_165insACAGCA, c.1760C > T in *POLG*, c.1291G > A in *COL5A1* and c.10242G > T in *ZNF469* were found to qualify the criteria for pathogenic mutations according to ACMG guidelines with PVS1 (pathogenic very strong) and multiple PP1 (probably pathogenic) evidence. Among these, different mutations in the *POLG* gene were found in two patients, respectively, and the two patients (patients 115 and 116) were not related (Supplementary Table 6).

### Neuropsychiatric disorders genes located in regions of high myopia loci

**Figure 7:**
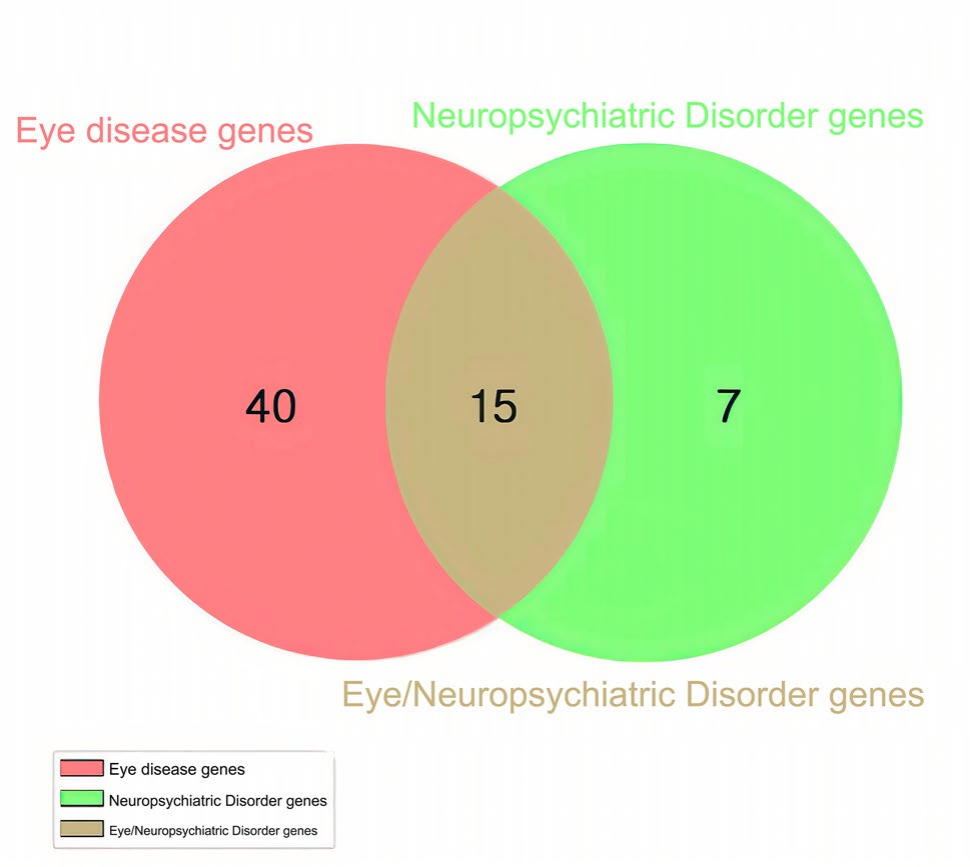
Venn diagrams of genes involved in eye and neuropsychiatric diseases. 15 genes were found to be associated with both ocular and neuropsychiatric diseases.

**Figure 8:**
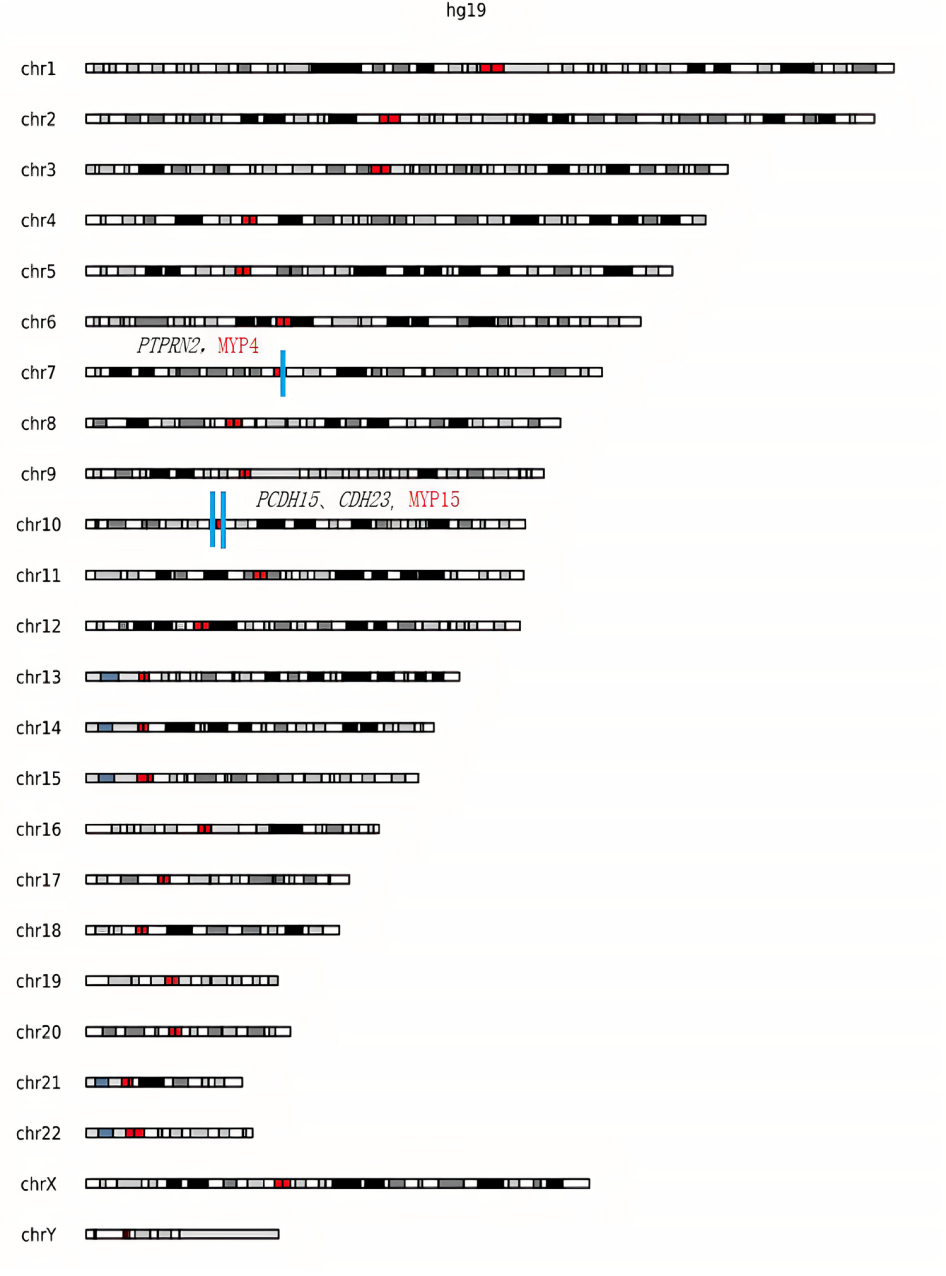
Peak chromosome distribution map. Red indicates the specific location of the neuropsychiatric disease gene on the chromosome, and blue indicates the partial gene position where the neuropsychiatric disease gene and the HM locus overlap.

In this study, 62 genes related to ocular diseases and 22 genes related to neuropsychiatric disorders were identified; of these, 15 genes were found to be associated with both ocular and neuropsychiatric diseases (Fig. 7). The causative genes for neuropsychiatric disorders in HM patients were mainly associated with retinopathy, especially retinitis pigmentosa (RP), which involves neuroprotective factors and humoral immunity, and cone optic rod dystrophy, which may develop into RP. The *TRMT44* gene is associated with partial epilepsy with pericentric spikes, and the *NPHP1* is associated with Joubert's syndrome, which can alter the nuclei of the medulla oblongata in addition to the base of the pons, causing various neuropathological changes. Localization of neuropsychiatric disorders genes revealed that three of them fall in the loci of the HM gene, namely *PTPRN2, PCDH15,* and *CDH23* (Fig. 8). The *PCDH15* is located on chromosome 10q21.1, which coincides with the MYP15 region of the HM locus, and has been identified to be associated with the development of Usher syndrome, which is associated with intellectual disability, EEG abnormalities or schizophrenia, in addition to ocular symptoms. *CDH23* is located on chromosome 10q21.1 and is in the MYP15 region of the HM locus, like *PCDH15*, which is also associated with Usher syndrome and retinitis pigmentosa.

In addition to the above-mentioned genes that are located on the HM locus, there are also neuropsychiatric disorders genes that are located very close to the known HM locus regions. *PLK4* is located on chromosome 4q28.1, adjacent to the MYP11 (4q22-27) region, *POMT2* is on chromosome 14q24.3, similar to the MYP18 (14q22.1–24.2) region, and the *TRMT44* is positioned on 4p16.1, which is in the 4p16 region as MYP23 (4p16.3).

## Discussion

Studies have shown that patients with high myopia have structural changes in their brain white matter, which are important factors contributing to neuropsychiatric disorders^16,17^. In this study, causative genes for neuropsychiatric disorders were found to account for 22.69% of strongly pathogenic genes in patients with HM, which represents the highest proportion among disease types other than ocular diseases. From clinical imaging research to genetics, our study deepens the study of high myopia and neuropsychiatric diseases.

Seitler et al. proposed a hypothesis to explain the higher-than-normal incidence of anxiety and depression in HM patients. He suggested that myopia arises due to tension formed by the tightening of the extraocular muscles around the eye, and that the body develops a resistance mechanism to this tension causing myopia. Further, this tension leads to an interruption of the individual separation process, wherein myopic patients experience individual separation anxiety that leads to feelings of not being able to live a normal life^18^. In a study of 360 patients with visual impairment in France, Germany, and Italy, one third of the patients with visual impairment were found to be affected by anxiety or depression^19^. A study by Yokoi et al. indicated that 22.0%–25.9% of patients with HM may experience depression or anxiety^20^. These findings indicate the high prevalence of anxiety and depression in HM patients in different countries and regions. In this study, we found that neuropsychiatric disorder-related genes accounted for 22.69% of the strongly pathogenic genes. This is consistent with the findings of Yokoi et al^20^. Our findings support the association of HM with depression and anxiety disorders at the genetic level. Further in-depth studies are required to provide more robust evidence.

In a study of 112 myopic British patients, depression and anxiety in HM patients were found to increase the risk of other diseases, such as myocardial infarction and rheumatoid arthritis^21^. In our study, HM patient 102 had concomitant symptoms of arthritis. Highly-pathogenic predicted genes *B3GALNT2* and *C5orf42* were identified in this patient, which are associated with brain abnormalities that can cause a variety of neuropathic alterations, including abnormal cerebellar development (an abnormality that includes both dysplasia and hypoplasia scenarios). In addition, *B3GALNT2* and *C5orf42* can also cause pathological changes in the nucleus accumbens. Whether the above genes play a role in the development of chronic arthritis deserves further investigation. Moreover, our findings provide genetic evidence for the inference of Rose et al.^21^.

The prevalence of seasonal affective disorder (SAD) in the healthy population is 1.5% to 9%^22^. Polish scholars first reported in 2016 that the prevalence of SAD in the visually impaired group was significantly higher than that in the control group^23,24^. Furthermore, The increase in ocular growth and the occurrence of SAD may be caused by inappropriate exposure to blue light^25,26^. We identified the *PCDH15* in HM patient 103, which is expressed in several tissues and organs of the body. PCDH15 is closely related to electroencephalogram abnormalities in neuropsychiatric diseases, intellectual disability, and the development of schizophrenia^27–29^. The genes *CDHR1, INPP5E, BBS1, SLC7A14* and *LRP5* which showed strong interactions with high myopia and neuropsychiatric disorders were identified in patients 90, 91, 93 and 96, respectively, all of which have an impact on retinal nutrition and function, thus supporting the hypothesis that retinal dysfunction may play a role in the pathogenesis of some cases of SAD^30^.

Ong et al. found that in the elderly population, myopic patients were twice as likely to develop cognitive dysfunction compared to normal individuals^31^. This suggests that myopia may be a major contributing factor in the development of cognitive dysfunction. Some results examining this association suggest a possible correlation between cognitive function and myopia^32–34^. A large cross-sectional study of 1,022,425 Israeli adolescents demonstrated a strong association between cognitive function and myopia^35^. One hypothesis is based on the biological relationship between myopia and cognitive function, suggesting that myopia arises due to over development of the eye, which itself is correlated with the brain, and thus leads to higher IQ in myopic individuals^36,37^. Another hypothesis suggests that there is a pleiotropic relationship between cognitive function and myopia. For example, brain disorders and eye disorders are affected by the same gene or set of genes at the same time^38,39^. Our study identified 62 causative genes for ocular diseases and 22 causative genes for neuropsychiatric disorders among the most pathogenic genes in HM patients. Of these, 15 genes are expressed in both the eye and brain, namely *SORBS3, FKRP, SOX5, PLCH2, TRMT44, POMT2, PCDH15, PLK4, ARL13B, BBS1 B3GALNT2, CDH23, BBS9, SALL4, FKRP.* This finding provides new evidence to support the second hypothesis that brain and eye diseases may be affected by the same gene or group of genes at the same time. In addition, the association of some neuropsychiatric disorders genes with HM phenotypes has not yet been clarified, but these genes are localized in the locus region of the HM gene and their positioning on the chromosome exhibits absolute positional similarity. Therefore, further studies are required to investigate any potential functional interactions.

Resting-state functional magnetic resonance imaging analysis shows that the brains of HM patients exhibit low-frequency fluctuations and amplitude changes of the default mode network (DMN) that are different from healthy people, demonstrating the correlation between HM and cognitive changes^40^. In this study, a total of 22 genes in HM patients were identified as strongly pathogenic and expressed in the brain, 11 of which were adjacent to the MYP locus, *PTPRN2* on MYP4, *PCDH15* and *CDH23* on MYP15, *PLK4* immediately adjacent to the MYP11 region, *POMT2* near the MYP18 region, *TRMT44* is in the 4p16 interval as well as MYP23, and the *PLCH2, B3GALNT2, NPHP3, C5orf42* and *SORBS3* have corresponding high proximity loci nearby. Our finding may provide biological evidence for the above imaging scan results. According to Huang et al. structural brain dysfunction may result from HM accompanied by damage to the visual cortex^41^. Liu J et al. reveals a positive correlation between mGMV and c-fos and NeuN expression in visual cortex of form-deprivation myopia (FDM) rats, also suggesting the relationship between cortical activity and the structural plasticity in visual cortex^42^. Our study identified 15 genes expressed both in the eye and neuropsychiatric disorders, among which protein interactions between *PLCH2, BBS9, ARL13B*, and *BBS1* were evident. Further studies are required to determine whether these genes are associated with altered gray matter volume in the brain.

WES is an important tool for the detection of genetic disorders. It enables in-depth characterization of the relationship between specific genetic mutations and different diseases. However, due to the widespread genetic heterogeneity and clinical phenotypic heterogeneity, the understanding of both phenomena is still evolving. In this study, WES of patients with HM phenotype revealed a large proportion of neuropsychiatric disorders gene clusters in addition to ocular disease genes, which provides a new perspective to better understand the pathogenesis in patients with complex clinical phenotypes. Most previous studies have entailed biased analysis of WES data after identification of clinical phenotypes; however, comprehensive analysis of WES data can also facilitate the discovery of relevant complex clinical phenotypes. In particular, comprehensive analysis of WES data in a patient population with well-defined clinical phenotypes may reveal associations between different clinical phenotypes while exploring the disease itself. The two complement each other, ultimately forming a closed loop for accurate diagnosis of genetic diseases.

The study had some limitations. For one thing, this study lack of comprehensive clinic data from patients with HM, as an eye hospital without neuropsychiatric testing facilities, we cannot refer patients to other hospitals for neuropsychiatric testing if the patients report no relevant signs or symptoms. For another, the study is limited by the small sample size, although our synthesis-driven analysis of WES data has improved the accuracy of genetic pathogenicity in HM patients. Whether the genes we discovered act on both high myopia and neuropsychiatric diseases needs to be verified with a larger HM sample size. Coincidentally, 10 of 15 genes that act on both high myopia and neuropsychiatric diseases included *FKRP, POMT2, PCDH15, PLK4, ARL13B, BBS1, CDH23, BBS9, SALL4, FKRP* were also discovered in the WES data of high myopia population genetics study by Wan L et al.^43^. Additionally, *PCDH15, CDH23, BBS1,* and *BBS9* were simultaneously verified in the WES sequencing results of 325 high myopia patients in Guangdong, China, and *BBS1, BBS9* were verified again in the WES data of 6215 school-aged children with high myopia^44,45^.

In summary, we found Causative genes for neuropsychiatric disorders accounted for the high proportion of genes that exhibited high pathogenicity in HM patients, to our knowledge, this is the first study of genes interacting with HM and Neuropsychiatric Disorders in Northwest China. The findings presented three causative genes for neuropsychiatric diseases falled at the HM locus. These findings expand our knowledge of the WES data, and provide clues for further genetic study of complex diseases.

### Supplementary Information

Supplementary Tables.

## Data Availability

The datasets generated during and/or analysed during the current study are available from the corresponding author on reasonable request.
