# Supplementary material for: Screening of genes interacting with high myopia and neuropsychiatric disorders
Source: Sci Rep. 2023 Oct 26;13:18347. doi: 10.1038/s41598-023-45463-y (PMC10603034; doi:10.1038/s41598-023-45463-y)
Supplement: Supplementary file 1 — Supplementary Tables. [file 41598_2023_45463_MOESM1_ESM.zip › Supplementary-PDF/Supplementary Table 1.pdf]

Supplementary table1: Clinical data from 83 patients with high myopia

			BCVA		SE（D）		AL（mm）	
**patients ID**	**gender**	**Age at presentation(y)**	**OD**	**OS**	**OD**	**OS**	**OD**	**OS**
25	M	6	0.1	0.1	-7.00	-8.50	26.05	26.54
26	F	45	0.1	0.3	-21.00	-21.50	31.21	31.58
27	F	38	1.0	1.0	-15.75	-16.00	29.12	28.96
28	F	11	0.6	0.6	-10.25	-9.75	26.82	26.45
31	F	25	0.5	0.6	-16.25	-15.00	29.09	28.79
33	M	11	1.0	1.0	-7.50	-7.50	26.11	26.16
36	F	12	0.8	0.8	-7.50	-9.00	25.55	26.13
39	F	12	0.8	0.8	-7.50	-7.00	25.83	25.97
41	F	24	0.8	0.8	-12.25	-12.75	27.72	27.91
43	F	13	1.0	1.0	-7.00	-6.25	26.55	26.12
44	M	10	1.0	1.0	-5.00	-5.75	25.93	25.91
45	M	9	0.8	1.0-	-9.25	-9.00	26.37	26.21
46	M	13	1.0	1.0	-6.50	-6.75	26.36	26.46
47	F	11	1.0	1.0	-6.75	-6.50	26.27	26.07
48	F	11	0.8	0.8-	-8.50	-8.75	25.60	25.62
49	M	15	0.7	0.8	-9.50	-14.50	25.61	27.50
50	F	12	1.0	1.0	-10.25	-10.75	28.92	29.44
51	F	12	1.0	1.0	-10.75	-10.00	26.52	26.17
52	F	16	0.8	0.8-	-11.50	-9.50	26.89	26.25
53	M	7	0.3+	0.25+	-13.00	-11.25	26.71	26.09
54	F	12	1.0	1.0	-6.75	-7.25	25.34	25.51
55	F	14	0.8	0.8	-13.25	-11.75	26.60	25.59
56	M	16	0.7	0.6	-18.75	-18.00	29.68	29.21
57	M	16	0.8	0.8-	-14.00	-14.00	29.12	29.17
58	F	5	0.2	0.16	-15.50	-11.00	24.92	23.77
59	M	12	1.0	1.0	-8.50	-9.00	26.09	26.23
60	F	14	1.0	1.0	-6.75	-7.00	25.02	25.03
61	F	14	1.0	1.0	-9.75	-12.00	26.83	27.35
64	F	13	1.0	1.0	-12.50	-11.00	28.39	27.51
65	F	13	0.8	0.8	-7.00	-5.75	26.23	25.96
67	F	5	0.25	0.2	-11.50	-12.50	26.92	27.06
68	F	24	0.7	0.7	-13.50	-12.50	27.94	27.73
69	M	45	0.2	0.2	-26.00	-24.00	31.37	30.21
70	F	15	1.0	1.0	-11.25	-11.50	27.45	27.24
71	F	29	0.8	0.8	-18.50	-17.75	28.54	27.98
72	M	21	1.0	1.0	-7.00	-6.75	25.01	24.98
73	M	50	1.0	1.0	-6.00	-6.00	23.79	23.67
74	M	45	1.0	1.0	-6.00	-6.25	24.89	24.94
75	M	48	0.8	1.0	-6.50	-6.25	26.58	26.26

76	M	48	1.0	0.8	-9.50	-9.25	27.01	27.22
77	F	48	0.5	0.5	-11.00	-10.50	24.88	24.75
78	F	44	1.0	1.0	-0.75	-8.00	22.82	22.67
79	M	45	1.0	1.0	-2.50	-6.00	24.22	24.19
80	F	49	0.4	0.25	-13.75	-16.50	27.50	28.67
81	M	48	1.0	1.0	-6.00	-6.00	24.48	24.55
82	M	48	0.8	0.8	-4.00	-4.50	26.61	26.49
83	M	14	1.0	1.0	-6.00	-6.25	25.06	25.14
84	F	25	0.3	0.25	-19.50	-21.00	31.02	31.72
85	F	57	0.1	0.3	-23.75	-16.75	30.99	29.61
89	M	19	0.8	0.8	-9.75	-10.00	27.80	27.90
90	F	19	1.0	1.0	-7.75	-8.25	28.17	28.35
91	F	19	0.8	0.8	-8.75	-8.50	27.56	27.52
92	F	23	0.8	0.8	-8.75	-7.75	26.87	26.85
93	F	21	1.0	1.0	-10.00	-11.75	26.53	27.22
94	M	22	1.0	1.0	-8.50	-8.75	27.52	27.53
95	M	21	1.0	1.0	-8.50	-8.25	26.75	26.45
96	M	18	0.8	0.8	-9.50	-10.00	26.71	26.65
97	F	23	0.5	0.6	-16.25	-16.00	29.86	29.43
99	M	19	0.8	0.8	-9.25	-10.00	27.97	28.18
100	F	22	1.0	1.0	-7.25	-7.75	26.12	26.27
101	M	20	1.0	1.0	-12.00	-12.25	27.32	27.37
102	M	17	1.0	1.0	-9.50	-9.50	27.46	27.50
103	F	43	1.0	1.0	-6.25	-6.00	24.05	24.19
104	F	15	0.8	0.1	-6.50	-15.00	26.12	26.91
105	M	33	0.8	1.0	-10.00	-9.00	27.63	NA
106	F	27	1.0	1.0	-12.00	-12.00	27.24	27.33
107	F	20	0.8	1.0	-14.75	-14.50	26.94	27.33
109	M	19	1.0	1.0	-10.25	-9.25	27.75	27.14
110	F	24	1.0	1.0	-10.75	-9.50	26.79	26.51
111	M	18	1.0	1.0	-6.25	-6.50	26.65	26.76
113	F	21	1.0	1.0	-16.00	-14.25	26.64	26.91
114	M	18	1.0	1.0	-9.25	-8.50	27.16	26.85
116	M	20	0.8	0.8	-12.25	-17.50	27.96	28.17
120	F	15	1.0	1.0	-6.00	-6.00	23.94	23.94
121	M	42	1.0	1.0	-6.75	-6.00	24.63	23.91
123	M	41	1.0	1.0	-6.50	-6.75	25.79	25.74
124	F	41	1.0	1.0	-6.25	-6.25	26.47	26.64
125	M	54	1.0	1.0	-7.25	-7.75	25.86	26.05
126	F	22	1.0	1.0	-6.00	-6.00	25.62	25.45
127	F	14	1.0	1.0	-8.00	-7.75	25.25	25.10
128	F	11	0.8	0.6	-10.50	-10.50	25.26	25.23
129	M	44	1.0	1.0	-7.25	-7.75	25.96	26.15
130	F	43	1.0	1.0	-6.00	-6.50	22.78	22.67

F: female;M: male;BCVA: best corrected visual acuity; SE: refractive error; AL: axial length; NA:not available.
