# Supplementary material for: Screening of genes interacting with high myopia and neuropsychiatric disorders
Source: Sci Rep. 2023 Oct 26;13:18347. doi: 10.1038/s41598-023-45463-y (PMC10603034; doi:10.1038/s41598-023-45463-y)
Supplement: Supplementary file 1 — Supplementary Tables. [file 41598_2023_45463_MOESM1_ESM.zip › Supplementary-PDF/Supplementary Table 2.pdf]

Supplementary table2: the list of 37 novel mutations

Patients ID	Gene	Chr	Mutation Type
115	*API5*	11	nonsynonymous SNV
96	*ATAD3A*	1	nonsynonymous SNV
90	*BMP6*	6	nonframeshift deletion
90	*CDHR1*	10	nonsynonymous SNV
91	*CEP78*	9	nonsynonymous SNV
105	*COL1A1*	17	nonsynonymous SNV
97	*CYP1B1*	2	nonsynonymous SNV
97	*FKRP*	19	stopgain
113	*HPS5*	11	nonsynonymous SNV
112	*HTT*	4	frameshift deletion
111	*IDH3B*	20	nonsynonymous SNV
91	*INPP5E*	9	nonsynonymous SNV
109	*KCNA4*	11	nonsynonymous SNV
92	*KNDC1*	10	nonsynonymous SNV
112	*MAFB*	20	nonsynonymous SNV
99	*MED1*	17	nonsynonymous SNV
112	*NCOA2*	8	nonsynonymous SNV
103	*PCDH15*	10	nonsynonymous SNV
85	*PHF2*	9	nonsynonymous SNV
99	*PIKFYVE*	2	nonsynonymous SNV
99	*PKHD1*	6	nonsynonymous SNV
100	*PLCH2*	1	nonsynonymous SNV
109	*PLK4*	4	nonsynonymous SNV
92	*PNPT1*	2	nonsynonymous SNV
112	*POLG*	15	nonsynonymous SNV
102	*POMT2*	14	nonsynonymous SNV
105	*POMT2*	14	nonsynonymous SNV
112	*PZP*	12	nonsynonymous SNV
91	*SLC7A13*	8	nonsynonymous SNV
92	*SORBS3*	8	nonsynonymous SNV
99	*SOX5*	12	nonsynonymous SNV
114	*SPTBN1*	2	nonsynonymous SNV
90	*TGFBI*	5	nonsynonymous SNV
102	*TRMT44*	4	nonsynonymous SNV
103	*TRMT44*	4	nonsynonymous SNV
110	*TRPM1*	15	nonsynonymous SNV
114	*VAX1*	10	nonsynonymous SNV
