# Supplementary material for: Screening of genes interacting with high myopia and neuropsychiatric disorders
Source: Sci Rep. 2023 Oct 26;13:18347. doi: 10.1038/s41598-023-45463-y (PMC10603034; doi:10.1038/s41598-023-45463-y)
Supplement: Supplementary file 1 — Supplementary Tables. [file 41598_2023_45463_MOESM1_ESM.zip › Supplementary-PDF/Supplementary Table 3.pdf]

Supplementary table 3: The list of 72 rare mutations

Patients ID	Gene	Chr	Mutation Type
114	*ABCC6*	16	frameshift deletion
95	*ACADSB*	10	nonsynonymous SNV
114	*ADAMTSL1*	9	nonsynonymous SNV
94	*ALDH18A1*	10	nonsynonymous SNV
116	*AP5B1*	11	nonsynonymous SNV
91	*ARL13B*	3	nonsynonymous SNV
104	*ARSK*	5	nonsynonymous SNV
115	*ATP6V0A2*	12	nonsynonymous SNV
102	*B3GALNT2*	1	nonsynonymous SNV
93	*BBS1*	11	nonsynonymous SNV
97	*BBS2*	16	nonsynonymous SNV
115	*BBS9*	7	nonsynonymous SNV
101	*BICC1*	10	nonsynonymous SNV
112	*C5*	9	nonsynonymous SNV
102	*C5orf42*	5	nonsynonymous SNV
114	*CAMKMT*	2	nonsynonymous SNV
114	*CAPN5*	11	stopgain
115	*CASS4*	20	nonsynonymous SNV
109	*CDH23*	10	nonsynonymous SNV
103	*CNGB1*	16	nonsynonymous SNV
116	*COL4A5*	X	nonsynonymous SNV
115	*COL5A1*	9	nonsynonymous SNV
102	*COL9A1*	6	nonsynonymous SNV
100	*DPYD*	1	nonsynonymous SNV
101	*ECEL1*	2	nonsynonymous SNV
112	*FAM161A*	2	nonsynonymous SNV
116	*FKRP*	19	nonsynonymous SNV
104	*FOXL2*	3	nonsynonymous SNV
102	*FOXP4*	6	nonsynonymous SNV
111	*FRMD7*	X	nonsynonymous SNV
102	*GALK1*	17	nonsynonymous SNV
94	*GRIK1*	21	nonsynonymous SNV
114	*GRM6*	5	nonsynonymous SNV
95	*HGSNAT*	8	nonsynonymous SNV
110	*HMCN1*	1	nonsynonymous SNV
111	*JAG1*	20	nonsynonymous SNV
115	*LRIT1*	10	nonsynonymous SNV
109	*LRP5*	11	nonsynonymous SNV
96	*LRP5*	11	nonsynonymous SNV

114	*LTBP2*	14	nonsynonymous SNV
93	*MUT*	6	nonsynonymous SNV
110	*MYO5B*	18	nonsynonymous SNV
102	*MYOF*	10	nonsynonymous SNV
102	*NPHP1*	2	nonsynonymous SNV
114	*NPHP3*	3	stopgain
96	*NRIP1*	21	nonsynonymous SNV
115	*PDE11A*	2	frameshift deletion
116	*PEX1*	7	nonsynonymous SNV
101	*PKHD1*	6	nonsynonymous SNV
115	*POLG*	15	nonframeshift insertion
116	*POLG*	15	nonsynonymous SNV
114	*PRDM5*	4	nonsynonymous SNV
113	*PTPRN2*	7	nonsynonymous SNV
96	*PZP*	12	nonsynonymous SNV
115	*RFPL1*	22	stopgain
115	*RP1*	8	nonsynonymous SNV
115	*RYR1*	19	nonsynonymous SNV
115	*SALL4*	20	nonsynonymous SNV
110	*SEC23B*	20	nonsynonymous SNV
116	*SLC12A6*	15	nonsynonymous SNV
102	*SLC2A10*	20	nonsynonymous SNV
116	*SLC4A11*	20	nonframeshift deletion
95	*SLC7A14*	3	nonsynonymous SNV
93	*SMARCAL1*	2	nonsynonymous SNV
113	*SMARCC1*	3	nonsynonymous SNV
95	*SRPK2*	7	nonsynonymous SNV
101	*TJP2*	9	nonsynonymous SNV
95	*TRIT1*	1	nonsynonymous SNV
113	*USPL1*	13	nonsynonymous SNV
92	*ZEB1*	10	nonsynonymous SNV
92	*ZEB1*	10	nonsynonymous SNV
99	*ZNF469*	16	nonsynonymous SNV
