# Supplementary material for: Screening of genes interacting with high myopia and neuropsychiatric disorders
Source: Sci Rep. 2023 Oct 26;13:18347. doi: 10.1038/s41598-023-45463-y (PMC10603034; doi:10.1038/s41598-023-45463-y)
Supplement: Supplementary file 1 — Supplementary Tables. [file 41598_2023_45463_MOESM1_ESM.zip › Supplementary-PDF/Supplementary Table 4.pdf]

Supplementary table 4: Summary of Neuropsychiatric and Ocular Disease Genes in Novel Mutations

location	Patient ID	Gene	Exon	Transcript	Nucleotide	Protein	Genomad	Expression
10q23.1	90	*CDHR1*	16	NM_001171971	c.G1955C	p.W652S	NA	EYE
5q31.1	90	*TGFBI*	4	NM_000358	c.C423G	p.F141L	NA	EYE
9q21.2	91	*CEP78*	1	NM_001098802	c.G130T	p.D44Y	NA	EYE
9q34.3	91	*INPP5E*	6	NM_019892	c.C1364A	p.T455N	NA	EYE
3q11.1	91	*ARL13B*	3	NM_144996	c.A247G	p.I83V	0.0004575	EYE/Neuro
2p22.2	97	*CYP1B1*	2	NM_000104	c.T284C	p.V95A	NA	EYE
8p21.3	92	*SORBS3*	9	NM_001018003	c.T899C	p.M300T	NA	EYE/Neuro
11q13.2	93	*BBS1*	4	NM_024649	c.G163A	p.V55M	0.0002285	EYE/Neuro
19q13.32	97	*FKRP*	4	NM_001039885	c.C1449A	p.Y483X	NA	EYE/Neuro
12p12.1	99	*SOX5*	7	NM_178010	c.C874T	p.P292S	NA	EYE/Neuro
1p36.32	100	*PLCH2*	10	NM_001303012	c.A1331T	p.K444M	NA	EYE/Neuro
4p16.1	102	*TRMT44*	3	NM_152544	c.T947C	p.M316T	NA	EYE/Neuro
14q24.3	102	*POMT2*	9	NM_013382	c.C1072T	p.H358Y	NA	EYE/Neuro
11p14.1	109	*KCNA4*	2	NM_002233	c.G664T	p.D222Y	NA	EYE
15q13.3	110	*TRPM1*	21	NM_001252020	c.T2972G	p.L991R	NA	EYE
20p13	111	*IDH3B*	8	NM_001258384	c.A755G	p.N252S	NA	EYE
15q26.1	112	*POLG*	11	NM_001126131	c.A1976G	p.H659R	NA	EYE
11p15.1	113	*HPS5*	11	NM_007216	c.A1039G	p.S347G	NA	EYE
10q25.3	114	*VAX1*	3	NM_019892	c.C1364A	p.T455N	NA	EYE
