# Supplementary material for: Screening of genes interacting with high myopia and neuropsychiatric disorders
Source: Sci Rep. 2023 Oct 26;13:18347. doi: 10.1038/s41598-023-45463-y (PMC10603034; doi:10.1038/s41598-023-45463-y)
Supplement: Supplementary file 1 — Supplementary Tables. [file 41598_2023_45463_MOESM1_ESM.zip › Supplementary-PDF/Supplementary Table 5.pdf]

Supplementary table 5: Summary of Neuropsychiatric and Ocular Disease Genes in Rare Mutations

location	Patient ID	Gene	Exon	Transcript	Nucleotide	Protein	Genomad	Expression
1q42.3	102	*B3GALNT2*	1	NM_001277155	c.T20G	p.L7R	0.00001191	EYE/ Neuro
10p11.22	92	*ZEB1*	6	NM_001174093	c.C766T	p.R256C	0.00001196	EYE
10q21.1	103	*PCDH15*	20	NM_001142765	c.A2738T	p.D913V	NA	EYE/ Neuro
21q21.3	94	*GRIK1*	13	NM_175611	c.C2069T	p.T690I	0.000007971	Neuro
3q26.2	95	*SLC7A14*	3	NM_020949	c.G332A	p.R111Q	0.00001195	EYE
8p11.2	95	*HGSNAT*	4	NM_152419	c.C475T	p.P159S	0.0001004	EYE
11q13.2	96	*LRP5*	23	NM_001291902	c.G2900T	p.C967F	0.0002285	EYE
16q13	97	*BBS2*	12	NM_031885	c.A1514G	p.E505G	0.00003579	EYE
16q24.2	99	*ZNF469*	2	NM_001127464	c.G10242T	p.R3414S	0.000987	EYE
4q28.1	109	*PLK4*	4	NM_001190799	c.C856T	p.L286F	NA	EYE/ Neuro
2q13	102	*NPHP1*	18	NM_001128179	c.G1672C	p.E558Q	0.0001431	Neuro
5p13.2	102	*C5orf42*	34	NM_023073	c.C6905T	p.T2302M	0.001659	Neuro
6q13	102	*COL9A1*	5	NM_001851	c.C344T	p.T115M	0.0003113	EYE
17q25.1	102	*GALK1*	5	NM_000154	c.T617G	p.L206W	0.0001082	EYE
10q23.33	102	*MYOF*	16	NM_013451	c.C1346T	p.T449I	0.0001555	EYE
16q21	103	*CNGB1*	32	NM_001286130	c.C3379T	p.R1127W	0.0001603	EYE

3q22.3	104	*FOXL2*	1	NM_023067	c.C1045G	p.R349G	0.0003384	EYE
11q13.2	109	*LRP5*	23	NM_001291902	c.C2876T	p.T959M	0.0002067	EYE
10q21.1	109	*CDH23*	11	NM_001171930	c.G1090A	p.G364S	0.000329	EYE/ Neuro
1q25.3	110	*HMCN1*	57	NM_031935	c.G8815A	p.G2939S	0.006912	EYE
20p12.2	111	*JAG1*	10	NM_000214	c.G1270A	p.A424T	0.00002785	EYE
	111	*FRMD7*	12	NM_194277	c.A1493T	p.Y498F	0.0001149	EYE
2p15	112	*FAM161A*	3	NM_001201543	c.C916T	p.R306W	0.0001645	EYE
2p15	112	*C5*	36	NM_001735	c.C4432T	p.R1478W	0.0001433	EYE
7q36.3	113	*PTPRN2*	13	NM_130842	c.C2071T	p.R691C	0.00002493	Neuro
2p21	114	*CAMKMT*	2	NM_024766	c.G160A	p.D54N	0.00001598	Neuro
3q22.1	114	*NPHP3*	15	NM_153240	c.C2104T	p.R702X	0.00004773	Neuro
5q35.3	114	*GRM6*	7	NM_000843	c.C1405T	p.R469W	0.0002592	EYE
11q13.5	114	*CAPN5*	2	NM_004055	c.C157T	p.R53X	0.00004831	EYE
14q24.3	114	*LTBP2*	34	NM_000428	c.G4934A	p.R1645Q	0.0002604	EYE
4q27	114	*PRDM5*	3	NM_001300823	c.G182A	p.R61H	0.00001852	EYE
7p14.3	115	*BBS9*	17	NM_014451	c.G1913A	p.R638H	0.00002784	EYE/ Neuro
15q26.1	115	*POLG*	2	NM_001126131	c.164_165insACAGCA	p.Q55delinsQQQ	0.000000431	EYE
8q11.2	115	*RP1*	4	NM_006269	c.A4108G	p.K1370E	0.00002391	EYE

9q34.3	115	*COL5A1*	8	NM_000093	c.G1291A	p.G431R	0.00002009	EYE
10q23.1	115	*LRIT1*	4	NM_015613	c.G970A	p.G324R	0.00004454	EYE
19q13.2	115	*RYR1*	16	NM_000540	c.G1768T	p.D590Y	0.00006364	EYE
20q13.2	115	*SALL4*	2	NM_020436	c.G541A	p.V181M	0.0004455	EYE/ Neuro
19q13.32	116	*FKRP*	4	NM_001039885	c.C328T	p.R110W	0.0001042	EYE/ Neuro
15q14	116	*SLC12A6*	7	NM_001042497	c.C967T	p.R323C	0.0004455	Neuro
15q26.1	116	*POLG*	10	NM_001126131	c.C1760T	p.P587L	0.001545	EYE
20p13	116	*SLC4A11*	13	NM_032034	c.1633_1644del	p.545_548del	0.0001035	EYE
Xq22.3	116	*COL4A5*	33	NM_000495	c.G2858T	p.G953V	0.003602	EYE
