# Supplementary material for: Screening of genes interacting with high myopia and neuropsychiatric disorders
Source: Sci Rep. 2023 Oct 26;13:18347. doi: 10.1038/s41598-023-45463-y (PMC10603034; doi:10.1038/s41598-023-45463-y)
Supplement: Supplementary file 1 — Supplementary Tables. [file 41598_2023_45463_MOESM1_ESM.zip › Supplementary-PDF/Supplementary Table 6.pdf]

Supplementary table 6: Pathogenicity classification according to ACMG guidelines

Gene	mutation	amino acid change	ACMG	Evidence of pathogenicity
HMCN1	c.G8815A	p.G2939S	LB	PM1,PP3,BS1,BP1,BP6
SALL4	c.G541A	p.V181M	LB	PP3,BP1,BP6,PP1,PP4
CEP78	c.G130T	p.D44Y	LP	PM1,PM2,BP1,PP3，PP1,PP4
INPP5E	c.C1364A	p.T455N	LP	PM1,PM2,PP3,PP1,PP4
CYP1B1	c.T284C	p.V95A	LP	PM2,PP3,PP1,PP4
FKRP	c.C1449A	p.Y483X	LP	PM2,PP3,PP1,PP4
SOX5	c.C874T	p.P292S	LP	PM1,PM2,PP3,PP1,PP4
PLCH2	c.A1331T	p.K444M	LP	PM1,PM2,PP3,PP1,
POMT2	c.C1072T	p.H358Y	LP	PM1,PM2,PP3,PS2.PM7
PCDH15	c.A2738T	p.D913V	LP	PM1,PM2,BP1,PP3
KCNA4	c.G664T	p.D222Y	LP	PM1,PM2,PP2,PP3
ARL13B	c.A247G	p.I83V	LP	PM1,BS1,PP3,PP1,PP4
BBS1	c.G163A	p.V55M	LP	PM1,PM2,PP3,BP1,PP1,PP4
BBS2	c.A1514G	p.E505G	LP	PM1,PM2,PP3,BP1,PP1,PP4
B3GALNT2	c.T20G	p.L7R	LP	PS2,PM2,PP3
COL9A1	c.C344T	p.T115M	LP	PS2,PM2,PP5,BS2,BP1,PP3
GALK1	c.T617G	p.L206W	LP	PS2,PM1,PM2,PP3
MYOF	c.C1346T	p.T449I	LP	PP1,PP4,PM1,PP3
RP1	c.A4108G	p.K1370E	LP	PM2,BS2,BP1,PP3,PP1,PP4
LRIT1	c.G970A	p.G324R	LP	PM1,PP3,PP1,PP4
RYR1	c.G1768T	p.D590Y	LP	PM1,PM2,PP3,PP1,PP4
COL5A1	c.G1291A	p.G431R	P	PS1,PM2,PP3,PP1,PP4
POLG（patient ID:115）	c.164_165insACAGCA	p.Q55delinsQQQ	P	PVS1,PP3, PM7,PP1,PP4
POLG（patient ID:116）	c.C1760T	p.P587L	P	PVS1,PP3, PM7,PP1,PP4
ZNF469	c.G10242T	p.R3414S	P	PS1,PM1,BP4,BP6,PP3
